# Autoantibody Markers of Increased Risk of Malignancy in Patients with Dermatomyositis

**DOI:** 10.1007/s12016-022-08922-4

**Published:** 2022-02-11

**Authors:** Milena Marzęcka, Anna Niemczyk, Lidia Rudnicka

**Affiliations:** Department of Dermatology, Medical University of Warsaw, Warsaw, Poland

**Keywords:** Antinuclear antibodies, Cancer, Dermatomyositis, Malignancy

## Abstract

Dermatomyositis is a chronic inflammatory disease involving the skin and muscles. It most commonly occurs in adults with preponderance in females, but pediatric occurrence is also possible. The risk of malignancy in adult patients with dermatomyositis was reported to be 4.66-fold higher compared to that in the general population. A significantly increased risk of malignancy was reported within the first 12 months following the diagnosis of dermatomyositis (standardized incidence ratio equaled 17). One of the characteristic laboratory findings associated with dermatomyositis is the presence of circulating autoantibodies which are classified into two subgroups: myositis-specific and myositis-associated autoantibodies. It was shown that specific types of antibodies might be associated with an increased risk of malignancy. Current literature data indicate that the strongest correlation with malignant diseases was reported in anti-TIF1-γ-positive patients who were at a 9.37-fold higher risk of cancer. A 3.68-fold increase in the risk of cancer was also reported among patients with anti-NXP2 antibodies. Malignant diseases were reported in 14–57% of patients with anti-SAE antibodies. The presence of other autoantibodies may also be associated with an increased risk of malignancy. These data indicate that patients with circulating anti-TIF1-γ, anti-NXP2, and anti-SAE should be very closely monitored for dermatomyositis-associated malignant comorbidities. The aim of this review is to summarize the current data regarding the link between malignancy and the presence of specific antibodies in patients with dermatomyositis.

## Introduction

Dermatomyositis is a chronic, inflammatory disease that involves the skin and muscles. It most frequently occurs in adults aged 50–60 with preponderance in females, but pediatric occurrence (juvenile dermatomyositis) with the highest incidence between 5 and 15 years of age was also observed [1]. In addition to the classic adult and pediatric subtype of the disease, several other clinically important presentations, including paraneoplastic, drug-induced, hypomyopathic, and amyopathic variants, may be distinguished [2]. The disease is characterized by skeletal muscle weakness most commonly involving the proximal muscles of the upper and lower extremities and characteristic cutaneous lesions—flat-topped, erythematous to violaceous papules and plaques found over the metacarpophalangeal and both proximal and distal interphalangeal joints (formerly known as Gottron sign). Other typical dermatological features include bilateral lilac discoloration of the eyelids and eyelid area (heliotrope rash) and red, maculopapular well-demarcated rash on the chest wall (the V sign, shawl sign) [3]. The diagnosis of the disease is based on the presence of typical clinical symptoms and supported by the EULAR/ACR criteria [4]. Muscle biopsy is not required to confirm the diagnosis in patients with skin lesions typical of dermatomyositis [4].

The pathogenesis of dermatomyositis is multifactorial and still incompletely elucidated. The important role of genetic and environmental factors including infections, vitamin D deficiency, exposure to ultraviolet radiation, and drugs is emphasized. The literature indicates both autoimmune and nonimmune mediators in the pathogenesis of dermatomyositis. The particular role of autoimmune processes was suggested in the development of certain symptoms of the disease [5]. It is hypothesized that muscle damage is mediated by humoral factors (antibodies and the complement system) directed against the endothelial cells of endomysial capillaries. Two mechanisms are proposed for the induction of immune response. The first mechanism is associated with the overexpression of myositis-specific autoantigens in the inflamed muscles. The second mechanism is connected with the procurement of adjuvant activity by autoantigens [5]. Moreover, studies showed atypical signaling through the interferon pathway as an important pathological hallmark of the disease [6].

The presence of circulating autoantibodies is one of characteristic laboratory findings associated with autoimmunity in dermatomyositis. They may be detected in 40–80% of patients with the disease [1, 7–9]. They are classified into two subgroups: myositis-specific and myositis-associated autoantibodies [10]. Myositis-specific antibodies are detected in particular clinical syndromes within the spectrum of inflammatory myopathies, including dermatomyositis, polymyositis, and other rare subtypes such as necrotizing myopathy and inclusion body myositis [11]. Myositis-specific antibodies include antibodies directed against aminoacyl-tRNA synthetases (anti-Jo-1, anti-PL-7, anti-PL-12, anti-EJ, anti-OJ, anti-KS, anti-Ha, and anti-Zo), type 5 protein associated with melanoma (anti-MDA5), nuclear helicase (anti-Mi-2), signal recognition particle (anti-SRP), transcriptional intermediary factor 1 (anti-TIF1), nuclear matrix protein 2 (anti-NXP2), and SUMO-1 activating enzyme (anti-SAE) [1]. Studies revealed that the average incidence of myositis-specific antibodies amounted to 50% in dermatomyositis and polymyositis patients [12].

Anti-PM/Scl, anti-Ku, anti-Ro, anti-La, anti-U1-RNP, and anti-U3-RNP are the most common myositis-associated antibodies and may be found in 20% of patients with myositis [12]. They are also commonly detected in other systemic autoimmune diseases, mainly overlap syndromes [13].

As regards dermatomyositis, especially myositis-specific antibodies are clinically important biomarkers facilitating and supporting the correct diagnosis of the disease [14]. Their presence is associated with a characteristic clinical phenotype and may be useful in predicting and monitoring the disease as well as the response to treatment [15]. Idiopathic inflammatory myopathies are a group of diseases associated with an increased risk of malignancy. The latest literature data revealed that such an association was especially strong in patients with dermatomyositis [16]. Moreover, a higher incidence of malignancy was observed in males and in advanced-age patients with the disease [17]. The relationship between an increased risk of malignancy and the presence of certain disease-specific autoantibodies has been extensively studied in recent years. There is a hypothesis that there are mechanisms selecting the target antigens of MSA out of thousands of proteins in cells of patients. There is a possibility that the production of certain autoantibodies, especially cancer-associated autoantibodies, is triggered by the formation of cryptic epitopes resulting from a mutation of the target antigens, e.g., mutation in a gene encoding TIF-1 protein triggers production of anti-TIF1 antibodies. The cryptic epitopes which may be created by the changing of the pattern of protein disassembly as a result of amino acids mutations are presented on the surface of antigen-presenting cells by class I or II major histocompatibility complex (MHC). The crucial step toward triggering autoimmune response is recognizing the MHC-peptide complex as changed and impaired. Immunological tolerance to the epitopes may be incomplete and the cryptic epitopes expressed on antigen-presenting cells in some specific conditions may generate an autoimmune response [18].

The aim of this review is to summarize the current data regarding the link between malignancy and the presence of specific antibodies in patients with dermatomyositis.

## Dermatomyositis and Malignant Diseases

An association between dermatomyositis and malignancy was reported in numerous studies. The first case described in 1916 by Stertz was related to gastric cancer detected in a patient with idiopathic inflammatory myopathy [19]. The increased risk of cancer is associated with both dermatomyositis and other types of autoimmune myopathies. However, it is significantly higher in dermatomyositis with the incidence of malignancy ranging from 5.5 to 42% [20, 21]. A recent meta-analysis of 5 studies involving 4538 patients showed that the risk of cancer increased 4.66-fold in patients with the disease compared to the general population [17].

Malignant diseases may be diagnosed simultaneously, before or after the diagnosis of dermatomyositis. Malignant tumors are detected after the diagnosis of the disease in the vast majority of cases. The greatest risk of cancer was reported to occur within 12 months following the diagnosis of the disease (SIR of malignancy of 17.29). It is still increased 5 years after the diagnosis (SIR of malignancy of 1.37) [17].

A wide variety of cancers were reported in dermatomyositis. A significantly increased risk of cancer of lymphatic and hematopoietic system (SIR 22.72), lung (SIR 19.74), ovary (SIR 5.39), and no increased risk of stomach and prostate cancer was reported in a meta-analysis by Olazagasti et al. [22]. The location of neoplastic processes may depend on ethnicity, sex, and genetic background. According to the analysis of 2 large cohorts of patients with dermatomyositis, the most frequently reported malignancies were breast cancer (24.5%), hematologic malignancies (17.0%), colorectal cancers (9.4%), and prostate cancer (9.4%) [23]. Studies conducted in the Asian population showed that the nasopharynx was the most common location of malignancy with the incidence of 22.5–62.5% of all cancers [24, 25] (Table 1).

**Table 1:** Types of malignant diseases detected with the highest incidence in patients with dermatomyositis

Types of malignant diseases detected with the highest incidence in dermatomyositis	Percentage of certain types of malignant diseases in all patients with dermatomyositis and malignancy
Lymphatic and hematopoietic	3.6–17% [17, 23]
Lung	8–34% [17, 23]
Ovary	2–11.3% [17, 23]
Prostate	1–9.4% [17, 23]
Colon	5.2–14% [17, 23]
Breast	2.8–24.5% [17, 23]
Stomach	1–6% [17, 23]
Nasopharynx	22.5–62.5%* [24, 25]

*In Asian population

Numerous factors were also reported to be associated with a higher risk of cancer in patients with dermatomyositis. Although the disease occurs with female preponderance, a higher risk of malignancy was reported in males with a standardized incidence ratio (SIR) of cancer at 5.29 compared to 4.56 in females. The risk of malignancy was also higher in a group of patients beyond 44 years of age [17]. In contrast to adult patients, a higher risk of cancer was not found in patients with juvenile dermatomyositis [26]. In addition to older age and male sex, dysphagia, cutaneous necrosis, and some laboratory markers including accelerated erythrocyte sedimentation rate, increased C-reactive protein, or total creatine kinase concentrations were identified as the most important predictive factors of malignancy [16]. Conversely, the presence of interstitial lung disease, Raynaud’s phenomenon, and arthritis or arthralgia were associated with a lower risk of malignancy in dermatomyositis [27]. According to the literature, the meaningful role of certain specific autoantibodies was emphasized as factors associated with a higher risk of cancer in patients with dermatomyositis (Table 2). It is unclear if autoantibodies have a role in the pathogenesis of cancer [28]. They might be an epiphenomenon that arises in response to the impaired or mutant proteins of cancer cells [29]. Undoubtedly, autoantibodies may help in the early detection of malignancy [30].

**Table 2:** Prevalence of autoantibodies and percentage of patients with a certain type of autoantibodies who present with a malignant disease in dermatomyositis

Autoantibody	Target	Prevalence in dermatomyositis	Percentage of patients who present with a malignant disease
Anti-TIF1-γ	Transcription intermediary factor 1	7–41% [10]	19–100% [39]
Anti-NXP2	Nuclear matrix protein 2	2–25% [10]	24–43% [39, 46, 48]
Anti-SAE	Small ubiquitin-like modifier activating enzyme	1–10% [10]	14–57% [50–53]
Anti-ARS	Aminoacyl-tRNA synthetases	1–30% [1]	12% [55]
All types of autoantibodies	–	80% [1]	5.5–42% [20, 21]

## Antinuclear Antibodies Associated with an Increased Risk of Malignancy in Dermatomyositis

### Anti-TIF1 (Transcription Intermediary Factor 1) Antibodies

In 2006/2007, two independent research groups detected new antibodies directed against 140/155 kDa (anti-p140/p155) and 155 kDa (anti-p155) proteins in patients with dermatomyositis [31, 32]. Subsequent studies confirmed transcription intermediary factor 1-γ as the target of anti-p155 autoantibodies (anti-TIF1-γ) and transcription intermediary factor 1-ɑ was determined as the target of anti-p140 antibodies (anti-TIF1-ɑ) [33]. Moreover, samples of anti-p140/p155-positive sera reacted with TIF1-β which also belongs to the TIF1 protein family [33]. TIF1 molecules are composed of tripartite motif-containing proteins and they are involved in numerous cellular pathways such as DNA repair, transcription, chromatin regulation, mitosis, cell cycle control, immune mechanisms, and cell differentiation [34].

Anti-TIF1-γ antibodies in patients affected by dermatomyositis are associated with exacerbated skin symptoms including the presence of classic photodistributed rash, psoriasis-like lesions, hypopigmented and telangiectatic patches known as “red on white” patches, ovoid palatal patch, and palmar hyperkeratosis [35]. Moreover, anti-TIF1-γ positivity is associated with a hypomyopathic disease and an increased risk of gastrointestinal involvement [36]. In contrast, the presence of the antibodies is connected with the decreased risk of interstitial lung disease, calcinosis, Raynaud’s phenomenon, and arthralgias [37].

Anti-TIF1-γ positivity was identified as a cancer risk factor in dermatomyositis [38]. The incidence of malignancy in adult patients with dermatomyositis and antibodies to TIF-1γ ranged from 19 to 100% [39]. A recent meta-analysis by Best et al. included studies reporting data of 1962 patients with the disease. They indicated the presence of anti-TIF1-γ antibodies in 22.2% of patients. The study revealed that the risk of cancer was 9.37-fold higher in patients with anti-TIF1-γ. The pooled sensitivity and specificity of anti-TIF1-γ presence for diagnosing cancer in dermatomyositis was determined at 52% and 92%, respectively. A higher frequency of solid tumors than hematological malignancies was also noted in patients with anti-TIF1-γ antibodies [40]. Fujimoto et al. studied a large cohort of anti-TIF1-γ-positive patients and demonstrated that lung (29%), stomach (23%), breast (8%), and ovarian (8%) cancers were the most commonly detected [33]. Breast cancer (33%), ovarian cancer (19%), and lymphomas (14%) were the most frequent malignancies reported by Oldroyd et al. The study showed a significantly higher frequency of ovarian cancer in anti-TIF1-positive vs negative cases (19% vs 2%, respectively) [41]. Breast and ovarian malignancies were also reported as the most frequent in patients with dermatomyositis and anti-TIF1-γ antibodies in a recent study by Ikeda et al. [42]. Moreover, a study of Ogawa-Momohara et al. indicated that anti-TIF1-γ positivity was correlated with higher tumor staging compared to anti-TIF1-γ negativity in patients with dermatomyositis and cancer [43].

The risk of malignancy may depend on the time from diagnosis of the disease and the age of patients. A large cohort study conducted in the UK revealed that all malignancies were detected within 3 years before or 2.5 years following the diagnosis of dermatomyositis. Moreover, a significantly higher risk of cancer was observed in patients with dermatomyositis with anti-TIF1 aged above 39 years [41].

Fujimoto et al. reported a higher incidence of malignancy in patients affected by dermatomyositis and both anti-TIF1-γ and anti-TIF1-ɑ compared to patients with isolated TIF1-γ antibodies (73 vs 50%, respectively) [33]. The data were confirmed by Ogawa-Momohara et al. The frequency of cancer was also higher in patients with the co-occurrence of anti-TIF1-γ and anti-TIF1-ɑ compared to the group with isolated anti-TIF1-γ antibodies (70% vs 64%, respectively) [43]. Anti-TIF1-β was another antibody from the TIF-1 group detected in 2 patients with cancer-associated dermatomyositis. Ovarian cancer was diagnosed in both cases. However, this association requires additional research in a larger cohort [33].

### Anti-NXP-2 (Nuclear Matrix Protein-2) Antibodies

Anti-NXP-2 antibodies, also known as anti-MJ or anti-MORC3, were initially reported in the juvenile onset of the disease [44]. The NXP-2 protein is involved in the regulation of transcription and p53 protein activation [45].

As regards adult patients with dermatomyositis, anti-NXP-2 antibodies were detected with a wide variety of incidence ranging from 2 to 25% [10]. The presence of these antibodies was associated with an increased risk of peripheral edema, calcinosis, dysphagia, myalgia, and intestinal vasculopathy [46]. Conversely, anti-NXP-2 antibodies were negatively associated with interstitial lung disease [47].

The association between the presence of anti-NXP-2 and an increased risk of malignancy in patients with dermatomyositis was reported in several studies with the incidence of malignancy in NXP-2-positive patients ranging from 9 to 43% [39, 46, 48]. A Japanese study showed that a malignant tumor was detected in 43% (3 of 7) of patients affected by the disease and the presence of anti-NXP-2. All cancers were diagnosed at an advanced stage and all of them were also found in males. Cancer was most commonly detected within 1 year preceding or following the diagnosis of dermatomyositis [48]. The increased risk of cancer in anti-NXP-2-positive patients with dermatomyositis was confirmed by Fiorentino et al. in a group of 37 patients. A neoplastic disease was found in 24% of anti-NXP-2-positive patients with the odds ratio for the diagnosis of cancer of 2.5 (95% CI 1.0–6.1, *p* = 0.04). The study indicated that 83% of patients affected by dermatomyositis diagnosed with cancer had anti-TIF1-γ or anti-NXP-2 antibodies. The odds ratio for cancer development reached 4.7 (95% CI 1.7–12.8, *p* = 0.003) in a group of patients with one of those antibodies, NXP-2 or anti-TIF1-γ. Importantly, a higher frequency of malignancy was reported in males (83%) and patients over the age of 40. Multivariate analysis stratified by sex confirmed an increased risk of cancer in anti-NXP-2 patients with dermatomyositis only in males (OR 5.8 [95% CI 1.4–24.7]; *p* = 0.02) [39]. A recent analysis of Albayda et al. has shown a 3.68-fold increase in the risk of malignancy in anti-NXP-2-positive patients (95% CI 1.2–8.6) compared to the general population of the same age and sex [46].

### Anti-SAE (Small Ubiquitin-Like Modifier Activating Enzyme) Antibodies

Anti-SAE antibodies were detected in 2007 [49]. The targets of anti-SAE include A and B subunits of small ubiquitin-like modifier 1 (SUMO-1) activating enzyme. SAE is involved in the post-translational modification of proteins [18].

The prevalence of anti-SAE antibodies in dermatomyositis depends on ethnicity and ranges from 1 to 3% in the Asian and 5–10% in the European population [10]. These antibodies are rarely detected in juvenile type of dermatomyositis [10]. It was reported that anti-SAE positivity in patients affected by dermatomyositis was associated with the presence of classic photodistributed rash, dysphagia, cutaneous ulcers, and systemic symptoms such as fever or weight loss [50]. The presence of the antibodies was also connected with characteristic dark red or violaceous rash and pulmonary arterial hypertension [35].

The association between anti-SAE antibodies and malignancies in patients with dermatomyositis was suggested in several studies. Cancer was reported in 14–57% of patients with anti-SAE. However, the samples analyzed in studies were small (4–43 of anti-SAE-positive patients with dermatomyositis) [50–53]. As regards the Japanese cohort, anti-SAE was detected in 4.6% of patients and the study revealed a higher frequency of cancer in anti-SAE positive than in negative patients (4/7 vs 18/143, *p* < 0.0093). Rectum, uterus, esophagus, and colon cancers were detected in the studied group. The predominance of any particular cancer was not indicated [51]. Fujimoto et al. demonstrated a similar, high frequency of malignancy in the British and Japanese cohort in anti-SAE-positive patients affected by the disease (18% and 14%, respectively) [53]. The increased risk of malignancy associated with the presence of anti-SAE in patients with dermatomyositis has also been recently observed in a North American cohort. Malignancy was detected in 26% of anti-SAE-positive patients. Cancer was diagnosed in 2 cases within 12 months of the diagnosis of the disease. Three other cases of malignancies were detected within 5–7 years from the diagnosis of dermatomyositis [54]. The mentioned studies did not show the explicit link of anti-SAE presence and the short interval between diagnosis of dermatomyositis and the diagnosis of cancer. Thus, further research is warranted to clarify this correlation.

### Other Autoantibodies and Malignancy

Professional literature includes several reports of the existence of associations between cancer and other autoantibodies. Recent studies have shown an increased risk of malignancy in patients with myositis and anti-aminoacyl-tRNA synthetase antibodies (anti-ARS). Malignancy was diagnosed in 12% (*p* = 0.22) of a group of 165 anti-ARS-positive patients [55]. Ten out of 19 patients in this group were diagnosed with dermatomyositis and Jo-1 antibody was the most commonly detected in coexistence with malignancy (60% of cancer cases) [55]. In contrast to other previously mentioned autoantibodies which, in the majority of cases, were linked to the detection of cancer after the diagnosis of dermatomyositis, 50% of cancers in patients with anti-Jo-1 were diagnosed prior to and another half—simultaneously with the disease. Moreover, 9 out of 10 cancers were diagnosed in females and only one case in males. Breast cancer was detected with the highest frequency and the incidence of 30% of all detected malignancies [55].

In most of studies, lower prevalence of malignancy was reported in patients with anti-Mi-2 compared to individuals with other types of autoantibodies (3% vs 32%). However, a study by Hengstman et al. revealed a modestly higher incidence of cancer in patients affected by dermatomyositis with anti-Mi-2 antibodies (8%) [56]. Ceribelli et al. identified anti-Mi-2 in 30% of patients with dermatomyositis with cancer, although the studied sample was small (2/6) and the association did not reach statistical significance [57]. An increased risk of malignancy was reported in anti-Mi-2-positive patients in a recent study concerning a European cohort of idiopathic inflammatory myopathy patients (OR 2.06 [95% CI 1.16–3.63]) [8]. The association between anti-Mi-2 antibodies and an increased risk of malignancy in dermatomyositis remains controversial.

A trend toward increased malignancy (*p* = 0.15) was also reported in a group of patients with inflammatory myopathies and circulating autoantibodies against 3-hydroxy-3-methyl-glutaryl-coenzyme A reductase (anti-HMG-CoA). However, only 4% of those patients had a diagnosis of dermatomyositis. Therefore, studies in larger groups of patients with dermatomyositis are essential to confirm those reports [58].

Molina-Ruiz et al. reported the case of a 53-year-old male with dermatomyositis and anti-MDA5 antibodies. The patient had been diagnosed with thyroid cancer but this association requires further research [59].

## Conclusion

Cancer is one of the most common causes of mortality in patients with dermatomyositis [60]. Malignancies are detected after the diagnosis of the disease in the majority of cases [23]. Early cancer detection is essential for improving the prognosis of patients. Therefore, malignancy screening is crucial in dermatomyositis. Compared to the general population, the risk of malignancy in patients with dermatomyositis is 4.66-fold higher with an even higher risk in males and patients over 44 years of age [17]. A significantly increased risk of malignancy (SIR 17) was reported within the first 12 months after the diagnosis of the disease [17]. The detection of some specific antibodies may be an important tool in the identification of patients with dermatomyositis at a high risk of developing malignancy. Anti-TIF1-γ antibodies, especially co-occurring with anti-TIF1-ɑ and anti-NXP-2 antibodies have the strongest association with the development of neoplasms in patients with the disease. The presence of anti-SAE and other autoantibodies may also be correlated with an increased risk of cancer. Nevertheless, future research is essential to confirm the reports.

## Data Availability

The datasets generated during and/or analyzed during the current study are available from the corresponding author on reasonable request.
